# Pedunculated Focal Nodular Hyperplasia: When in Doubt, Should We Cut It Out?

**DOI:** 10.3390/jcm12186034

**Published:** 2023-09-18

**Authors:** Christos Tsalikidis, Athanasia Mitsala, George Pappas-Gogos, Konstantinos Romanidis, Alexandra K. Tsaroucha, Michail Pitiakoudis

**Affiliations:** 1 Second Department of Surgery, University General Hospital of Alexandroupolis, Democritus University of Thrace Medical School, 68100 Alexandroupolis, Greece; ctsaliki@med.duth.gr (C.T.); nancymits20@gmail.com (A.M.); pappasg8@gmail.com (G.P.-G.); kromanidismed@gmail.com (K.R.); pterion_ts@yahoo.gr (M.P.); 2 Laboratory of Experimental Surgery & Surgical Research, Democritus University of Thrace Medical School, 68100 Alexandroupolis, Greece

**Keywords:** focal nodular hyperplasia, pedunculated FNH, exophytic FNH, hepatic tumor, management

## Abstract

Focal nodular hyperplasia (FNH) is the second most common benign hepatic tumor and can rarely present as an exophytic solitary mass attached to the liver by a stalk. Most FNH cases are usually detected as incidental findings during surgery, imaging or physical examination and have a high female predominance. However, the pedunculated forms of FNH are particularly rare and commonly associated with severe complications and diagnostic challenges. Hence, our study aims to provide a comprehensive summary of the available data on the pedunculated FNH cases among adults and children. Furthermore, we will highlight the role of different therapeutic options in treating this clinical entity. The use of imaging techniques is considered a significant addition to the diagnostic toolbox. Regarding the optimal treatment strategy, the main indications for surgery were the presence of symptoms, diagnostic uncertainty and increased risk of complications, based on the current literature. Herein, we also propose a management algorithm for patients with suspected FNH lesions. Therefore, a high index of suspicion and awareness of this pathology and its life-threatening complications, as an uncommon etiology of acute abdomen, is of utmost importance in order to achieve better clinical outcomes.

## 1. Introduction

Focal nodular hyperplasia (FNH) is the second most common benign hepatic tumor and appears predominantly in women of childbearing age [1,2]. However, the pedunculated forms of FNH are particularly rare and usually associated with severe complications and challenges in differential diagnosis [3,4]. At this point, the exact etiology of FNH still remains unknown. Prior research suggested that FNH represents a regenerative hyperplastic response to the presence of preexisting hepatic circulatory abnormalities [2,5]. In fact, these benign lesions are commonly associated with vascular malformations, chemotherapy or a history of malignancy among adults and children [6,7,8,9].

In most cases, FNH is macroscopically described as a well-circumscribed, nodular solitary mass with a central stellate fibrous scar and peripherally radiating fibrous septa [10]. Even though an association between the long-term use of oral contraceptives and the growth of these lesions has been speculated, previous studies have demonstrated that such an etiologic relationship has not been established yet [11]. Interestingly, although the majority of FNH patients remain asymptomatic, life-threatening complications may still occur. In fact, pedunculated FNH cases are more commonly associated with clinical symptoms and complications [3].

To date, the use of imaging techniques is regarded as a significant and valuable addition to the diagnostic toolbox. The differentiation of FNH from other potentially malignant lesions of the liver is vital due to different treatment options. According to several previous studies, most researchers suggested follow-up imaging, eventually for a specific time period, or watchful waiting in uncomplicated cases with a definitive diagnosis of FNH [12]. Meanwhile, surgical intervention or minimally invasive procedures were considered the preferred treatment options in patients presenting with clinical manifestations and complications. Based on the literature, other surgical indications included cases with suspicious lesions for malignancy or diagnostic uncertainty and, eventually, progressive tumor growth [3,12,13,14].

It is worth mentioning that surgeons should deeply understand the advantages of laparoscopic surgery over traditional open surgery and the balance between the expected benefits and risks of the recommended therapeutic strategies. Our narrative review herein aims to provide a comprehensive summary of the collected data on the pedunculated forms of FNH among the adult and pediatric population. Based on the current literature, we will explore and discuss the epidemiology, pathophysiology and diagnostic workup of FNH, and provide new insights into the role of surgical and minimally invasive procedures in treating this pathology when necessary. Herein, we also propose a management algorithm for patients with suspected FNH lesions. Overall, a high index of suspicion and awareness of this clinical entity as a rare cause of acute abdomen is particularly essential in order to provide prompt intervention and improve patient safety. 

## 2. Current Landscape of Pedunculated Lesions in FNH

We performed a comprehensive online search of the MEDLINE/PubMed and Semantic Scholar databases, applying the search terms “Focal nodular hyperplasia”, “Pedunculated FNH”, and “Exophytic FNH”. The reference lists of the included articles were further investigated to identify additional relevant reports. Despite the presence or absence of clinical manifestations, all studies that focused on pedunculated or exophytic FNH cases in adult and pediatric patients were eligible for inclusion in this review. Regardless of the age, the sample size, and the study type, the current review analyzed studies with a definitive diagnosis of pedunculated FNH through imaging or histopathological examination.

The data extracted from the reviewed publications included the year of study, patient age, gender, size of the pedunculated or exophytic mass, presence of symptoms, therapeutic approach, and location of the tumor. Published studies that neither confirmed FNH diagnosis nor described exophytic and pedunculated forms of FNH were excluded from this analysis. Afterwards, based on the current literature on all FNH lesions, we explored and discussed the pathophysiology and diagnostic methods used for suspected FNH. The pedunculated forms differ from other FNH tumors in morphology (i.e., attached to the liver by a stalk), location (i.e., extrahepatic region), epidemiology, and increased risk of severe clinical manifestations, complications, and diagnostic challenges.

In 2001, Bader et al. [15] reported an exophytic mass measuring 4.5 cm and arising from liver segment V. The patient was treated through surgical resection due to clinical symptoms. A few years later, Byrnes et al. [16] performed segmental hepatectomy to treat a female patient with two pedunculated FNH tumors measuring 16 × 18 cm (segment IVb) and 3.5 × 3 cm (segment III), causing clinical manifestations. Leone et al. [17] presented a case of a woman with a pedunculated FNH mass of 6 cm attached to segment VI. Follow-up was recommended, as the patient remained asymptomatic.

Another research group reported a case of a lobulated 3.2 × 2.4 × 2.4 cm tumor arising from segment IVb, which was laparoscopically resected due to persistent symptoms [18]. In 2011, after embolization, Khan et al. [19] performed surgical resection of segments III and IVb, as two large symptomatic tumors arose from these segments in a female patient. In another study, Terada et al. [20] presented a case with a projected FNH tumor arising from the left liver lobe, incorrectly diagnosed preoperatively as a gastrointestinal stromal tumor through cross-sectional imaging.

Interestingly, Eris et al. [13] showed an uncommon presentation of exophytic FNH causing gastric outlet obstruction. In addition to the report of a male patient with symptoms related to a pedunculated FNH lesion by Dreizin et al. [21], another research team also aimed at investigating a case of a female patient with symptomatic FNH, which was laparoscopically resected [22]. Reddy et al. [23] presented another patient with FNH tumor, masquerading as a gastrointestinal stromal tumor detected by endoscopic ultrasound.

To date, several authors have reported pedunculated cases of FNH, while most of them were associated with clinical symptoms that required intervention [24,25,26,27,28,29,30,31]. Recently, Akaguma et al. [31] successfully performed laparoscopic surgery for pedunculated FNH in a female patient during pregnancy due to the increased risk of developing complications and severe clinical manifestations. Thus far, some pediatric cases with pedunculated FNH have also been reported in the literature [32,33,34,35]. All consecutive pedunculated or exophytic FNH cases in the literature review were identified and further investigated (Table 1 and Table 2).

In adults, the mean age ± standard deviation (SD) at presentation was 35.4 ± 9.46 years old (range 23–61 years), with a female/male ratio of 7.5:1. In children, the mean age ± SD at presentation was 9.6 ± 3.98 years old (range 3–13 years), with a female/male ratio of 4:1. According to several previous studies, this rare pathology affects primarily women of reproductive age and has a high female predominance. Our results revealed that these pedunculated or exophytic tumors are commonly larger than 3 cm in diameter, eventually reaching up to 18 cm. Regarding the clinical symptoms, most patients with pedunculated FNH lesions (66.7% in adults, 80% in children) presented with acute abdominal pain, nausea, vomiting, abdominal distention or a tender palpable abdominal mass. Indeed, only one pediatric patient (20%) and six adult patients (33.3%) remained asymptomatic, while half of the male adult patients also presented with clinical manifestations. 

**Figure 1:**
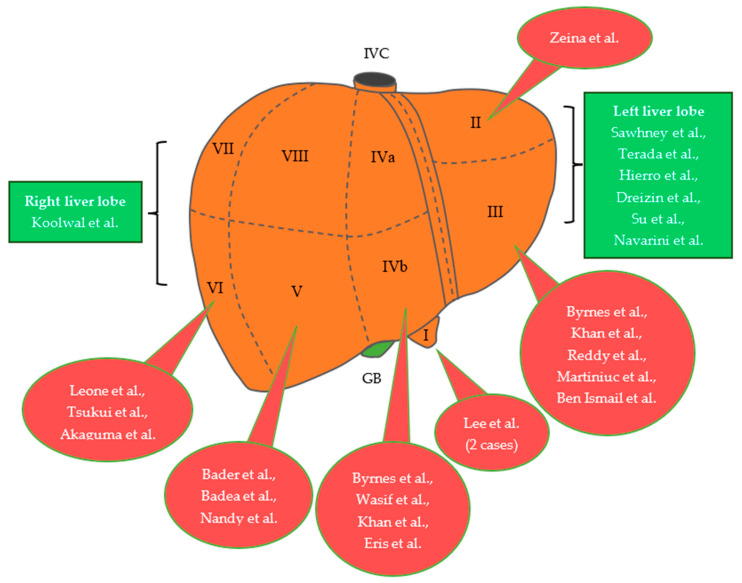
The segmental distribution of pedunculated or exophytic FNH cases for the adult and pediatric population, based on the literature review [13,15,16,17,18,19,20,21,22,23,24,25,26,27,28,29,30,31,32,33,34,35]. IVC, inferior vena cava; GB, gallbladder; FNH, focal nodular hyperplasia.

Furthermore, these pedunculated or exophytic tumors may arise from every segment of the liver (Figure 1). One case was managed conservatively with a watchful waiting approach, and two other patients underwent surgical resection following transarterial embolization (TAE). Surgery was performed in the majority of the cases, namely 16 and 5 adult and pediatric patients, respectively. Based on the literature, the main indications for surgical treatment were severe clinical manifestations, diagnostic uncertainty, and the increased risk of developing complications. 

## 3. Epidemiology

Focal nodular hyperplasia (FNH), which was first described by pathologist Hugh Edmondson in 1958 [36], represents the second most common benign solid lesion of the liver after hemangioma [1]. This pathology affects up to 3% of the general population and constitutes nearly 8% of all primary hepatic tumors [37]. In fact, all FNH lesions, including the pedunculated forms, predominate in women (female/male ratio: 8/1 to 12/1) from age 20 to 50 years, even though some cases have been reported in both sexes and at all ages [2]. FNH accounts for 2 to 7% of all hepatic tumors in children, with the median age at diagnosis being 8.7 years old [38,39,40,41,42]. However, contrary to other FNH lesions, the pedunculated forms of FNH are particularly rare, representing approximately 9% of the total FNH cases [4]. In fact, such rare forms differ from other FNH tumors in their morphology and location. Indeed, the lesions are connected to the liver by a stalk extending beyond the border of the liver. Therefore, pedunculated FNH is more frequently correlated with diagnostic challenges, as the thin pedicle usually remains undetected using different imaging techniques [3,4]. Interestingly, most cases are associated with severe and life-threatening complications, commonly mandating operative treatment [3,4].

## 4. Macroscopic and Microscopic Findings

**Figure 2:**
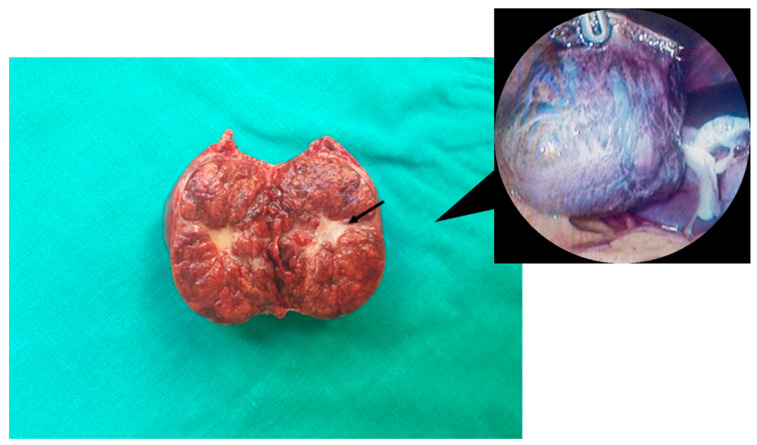
A 31-year-old female with pedunculated FNH arising from segment III of the liver. Gross appearance of the resected and divided solitary mass with the central stellate fibrous scar (black arrow), along with an intraoperative image showing the resected exophytic mass (4.5 × 3.8 × 2.8 cm), which was attached to the liver through a rotated vascular pedicle. Preoperatively, the patient presented to the emergency department with a sudden onset of severe abdominal pain, mainly located in the epigastrium and right hypochondriac region, without any nausea, vomiting or fever. Considering the clinical manifestations and imaging findings, emergency laparoscopic resection of the tumor was successfully performed in our department. The patient data were fully anonymized without using any identifiable personal details and written informed consent was also obtained. FNH, focal nodular hyperplasia.

Macroscopically, despite the presence of the pedicle in the pedunculated lesions, exophytic FNH (as well as most FNH tumors) is commonly characterized as a nodular solitary mass with well-circumscribed margins. It usually presents as a tan to yellow-brown, sometimes lobulated, mass with peripherally radiating fibrous septa and a central stellate scar-like fibrous connective tissue area [10,43] (Figure 2). The central scar is considered to be a pathognomonic feature of FNH, even though it is reported in up to 50% of all cases [10]. Overall, arterial hyperperfusion results in the activation of hepatic stellate cells, leading to the formation of the central scar [3]. FNH nodules typically have no surrounding capsule, while calcification is rarely observed (1-2%) in FNH cases [43,44]. Atypical exophytic FNH characteristics may include the presence of a pseudocapsule [37]. They are predominantly located in the right lobe or lateral segment of the left liver lobe. These benign pedunculated lesions are usually < 6 cm in diameter, ranging up to 18 cm (as shown in Table 1 and Table 2), and are commonly accompanied by a hypertrophic central feeding artery [1,10,31,43]. 

Histologically, pedunculated FNH could be characterized as either classic or atypical FNH based on the microscopic features of the tumors. The lack of normal nodular architecture, the presence of a central scar with peripherally radiating septa, areas of intramodular bile duct proliferation and malformed vessels are typical histologic findings of classic FNH [45,46,47]. Apart from the presence of cholangiolar proliferation, the lack of the aforementioned characteristic features is regarded as atypical or non-classic FNH (approximately 20% of cases), commonly associated with difficulties in differential diagnosis from other hepatic lesions [10]. In addition, both classic and non-classic FNH types contain various Kupffer cell concentrations [48]. In FNH cases, a characteristic map-like pattern is produced with immunostaining of glutamine synthetase, establishing itself as a valuable marker for distinguishing FNH from other liver lesions [49,50].

The atypical or non-classic FNH type includes the telangiectatic FNH, FNH with cytologic atypia, and mixed hyperplastic and adenomatous FNH. The telangiectatic form contains atrophic hepatic plates with dilated sinusoids. In contrast, the second form consists of large cell dysplasia areas, and the third one resembles either the telangiectatic form or hepatocellular adenomas [3].

## 5. Pathogenesis, Molecular Features and Associated Diseases

To date, the exact pathogenic mechanisms of exophytic FNH remain unclear. Compared to other FNH lesions, these exophytic tumors commonly share the same possible underlying mechanisms, molecular features, and relationships with specific diseases. Previously, FNH was thought to be a pedunculated adenoma, hamartoma, solitary hyperplastic nodule or focal cirrhosis; nevertheless, most authors currently support that FNH develops as a regenerative, hyperplastic response to the presence of congenital or acquired vascular malformations [2,5]. There are several observations that reinforce the theory that vascular abnormalities may play a pivotal role in the formation of most FNH cases, pedunculated or not. Recent research has demonstrated a significant association between the development of FNH and chemotherapy-induced hepatic circulatory abnormalities [6,7,51,52]. As reported in the literature, oxaliplatin-based treatments are usually correlated with sinusoidal obstruction, eventually accompanied by perisinusoidal fibrosis, veno-occlusive lesions and nodular regenerative hyperplasia [6,7]. 

In addition, FNH may also appear to be closely linked to several conditions associated with vascular abnormalities, including hepatic hemangiomas [53], hereditary hemorrhagic telangiectasia (Osler-Weber-Rendu syndrome) [54], congenital absence of the portal vein [55,56] and Budd-Chiari syndrome [57], and also cerebrovascular anomalies [58]. In another study, Libbrecht et al. [59] reported that there was a significant correlation between the presence of esophageal varices and FNH-like nodules, indicating that vascular alterations due to portal hypertension may lead to the formation of such nodular hyperplastic lesions.

Even though FNH is rarely described in children, most cases have been observed in pediatric malignancies treated with chemotherapy or Hematopoietic Stem Cell Transplantation (HSCT) [8,9,60]. In fact, several researchers suggested that vascular injuries due to circulatory disturbances, commonly induced by the abovementioned treatments, may contribute to the development of FNH [9,61]. However, there have also been a few reports of FNH cases in otherwise healthy individuals without the presence of risk factors or vascular abnormalities [35]. 

A few reports have shown that pregnancy and long-term use of oral contraceptives may lead to larger nodules in size and clinical manifestations, indicating a possible association between FNH and estrogen levels [62,63,64,65]. Indeed, a research team demonstrated that most FNH lesions showed estrogen receptor expression [66]. However, contrary to these findings, several studies supported that pregnancy or oral contraceptive use was found to be not significant for the development of FNH and its complications [3,11,67]. Thus far, such an etiologic relationship has not been established. Whether pregnancy or oral contraception could be related to the progression of FNH remains a subject of heated debate and requires further data collection and investigation [2,3,68,69].

It is worth mentioning that the co-occurrence of FNH and hepatocellular adenomas or carcinomas has been described in the literature [70,71,72,73,74]. Despite FNH being a rare condition, Langrehr et al. [71] suggested that a high index of suspicion and awareness should be maintained for rapidly growing FNH lesions, considering there are a few patients with hepatocellular carcinomas associated with FNH. Furthermore, Ercan et al. [72] studied a patient with hepatocellular carcinoma and FNH coexistence, revealing a clonal relationship between them and indicating the malignant potential of FNH. Interestingly, several authors have supported that certain subtypes of FNH are associated with the potential for malignant transformation [75,76]. Most telangiectatic FNH lesions are monoclonal and closely resemble hepatocellular adenomas.

In FNH, clonal analysis using the human androgen receptor (HUMARA) test revealed the reactive polyclonal nature of liver cells in 50–100% of the cases [77,78]. Previous research demonstrated altered messenger RNA (mRNA) expression levels of the angiopoietin genes (ANGPT1 and ANGPT2), which play a key role in blood vessel maturation. In fact, the ANGPT1/ANGPT2 ratio was found to be elevated among all FNH cases [78,79]. So far, most studies highlight and support the significant impact of vascular alterations on FNH pathogenesis.

Based on the mutation and loss of heterozygosity analysis, prior research revealed that the p53 gene was not related to FNH development [80]. The researchers showed that β-catenin, a multifunctional protein and a core component of the Wnt signaling pathway, may significantly affect the development of hepatocellular adenomas rather than FNH [80]. Most authors supported the polyclonal origin of FNH, indicating that these lesions are benign. Nevertheless, according to a few studies, monoclonality was also described, indicating a possible neoplastic process in some cases [75,77,81,82,83,84]. Other authors suggested that the whole FNH lesions are polyclonal, but some nodules of altered hepatocytes derived from these lesions are monoclonal, showing potential for malignant transformation [82,83]. 

## 6. Clinical Manifestations and Diagnostic Workup

The majority of FNH cases are asymptomatic and usually detected as incidental findings during physical and imaging examinations or surgical procedures. However, pedunculated FNH may sometimes present with acute abdominal pain, dyspepsia, epigastric discomfort, nausea, vomiting or a tender palpable abdominal mass [19,29]. In addition, alpha-fetoprotein (AFP) levels and liver function tests are generally within normal limits [10,85]. Slightly increased gamma-glutamyl transferase levels have been observed in some patients [43,86]. Overall, abnormal liver function tests have been reported in selected cases associated with external compression of hepatic vascular structures and intrahepatic or extrahepatic bile ducts [3]. 

Similar to other FNH lesions, the complications of pedunculated FNH include spontaneous infarction, rupture, and intra-abdominal hemorrhage [24,29,87]. Considerable risk of bleeding in pedunculated FNH cases may be related to preceding traumatic events [24]. Less common complications include compression of the adjacent vessels (e.g., portal vein obstruction) and organs [13,24,29,30,88,89]. Gastric outlet obstruction as a result of external compression has been reported in the existing literature [13]. Indeed, compression of surrounding organs and hemorrhage due to feeding vessel rupture represent serious complications of pedunculated FNH [29]. However, we should consider that pedunculated FNH lesions may additionally undergo torsion around their pedicle, eventually leading to infarction and severe clinical manifestations [28,29,34]. 

The challenges in the differential diagnosis of pedunculated FNH could lead to further diagnostic dilemmas. Even though several cases of FNH are accurately diagnosed, a prompt distinction between benign and malignant lesions is sometimes more of a gray area. Indeed, the differentiation between FNH and hepatocellular adenomas or carcinomas, which are tumors mostly requiring surgical intervention, may be challenging based on various imaging techniques. Such difficulties in the differential diagnosis are essential since benign and malignant lesions mostly mandate different approaches and treatment strategies. With the aim of accurately establishing a diagnosis when there are no typical findings of FNH, invasive diagnostic procedures may be deemed necessary.

Different imaging techniques can be used in order to diagnose pedunculated FNH [29]. Despite the fact that the mass is extended beyond the border of the liver and grown exophytically, pedunculated FNH is commonly characterized by imaging features similar to typical FNH [29]. Ultrasonography (US) is usually the first imaging modality used to evaluate hepatic lesions. Characteristic US features include a hypoechoic or isoechoic (and sometimes hyperechoic) mass along with displacement of surrounding vascular structures and a central hyperechoic scar, which can be visualized in only 20% of all FNH cases [43,90]. Color Doppler US could also help by detecting the basket pattern in some FNH patients and the typical stellate or spoke-wheel vascular pattern with vessels arising from a central feeding artery and radiating peripherally [43]. However, such vascular patterns may be absent in US imaging, leading to difficulties in making an accurate diagnosis and establishing the patients’ treatment plan [43]. 

In addition, contrast-enhanced ultrasound (CEUS) is also considered to be a valuable diagnostic tool for exophytic FNH. The presence of homogeneous enhancement and the early arterial centrifugal enhancement, eventually accompanied by a central feeding artery in the arterial phase, are typical findings commonly observed in FNH lesions [91]. Furthermore, these tumors appear with sustained enhancement in the portal venous and late phases, showing iso- or hyperenhancement compared to the surrounding liver parenchyma [91,92]. 

Thus far, several contradictory results have been reported regarding the association between the size of the lesions and the accuracy of CEUS in establishing FNH diagnosis. Some authors supported that the typical spoke-wheel pattern is more commonly noted in lesions larger than 3 cm [93,94,95]. In contrast, other researchers showed that the centrifugal filling pattern was more frequently observed in FNH lesions smaller than 3 cm [93,94,95,96,97]. In fact, the use of CEUS significantly increased the sensitivity and specificity for identifying FNH lesions smaller than 3.5 cm, reaching up to 93% and 100%, respectively [98,99]. Therefore, some researchers suggested using CEUS in diagnosing small lesions and magnetic resonance imaging (MRI) in diagnosing larger tumors (approximately > 3 cm) in size [97,98]. Nevertheless, it is worth mentioning that even though US is an easily accessible and radiation-free imaging modality, it is operator-dependent, and the image quality may be affected by issues related to patients’ obesity and bowel gas [3]. 

Prior to the administration of computed tomography (CT) intravenous contrast media, pedunculated FNH appears hypodense or isodense along with a hypodense central scar, which is observed in nearly one-third of total FNH cases [29,43,100]. Calcifications may be rarely found within the central scar in approximately 1% of all FNH patients [37,44]. During contrast-enhanced CT, pedunculated FNH lesions demonstrate rapid enhancement in the arterial phase along with the presence of the hypoattenuating central scar, visible in 60% of all cases [29,37,101]. These tumors appear isodense compared to the surrounding liver parenchyma in the portal venous phase. The central scar is visualized as hyperdense during the delayed phase due to the slow wash-out of the contrast agent [90,100]. Overall, some researchers supported that the central scar is more commonly noted in large FNH lesions than in small ones on CT imaging [37,102]. 

Even though the central scar is described as a typical finding in pedunculated FNH (as well as other typical FNH cases), this feature may also be present in large hemangiomas, fibrolamellar hepatocellular carcinomas and rarely in peripheral cholangiocarcinomas and liver metastases [103]. In addition, telangiectatic FNH may appear on CT scans with different features from those frequently observed in the classic FNH, considered by most authors as a variant of hepatocellular adenomas [3,104]. Difficulties differentiating FNH from fibrolamellar hepatocellular carcinomas may also occur in the case of atypical FNH findings detected by CT, as both tumors can be found in patients with similar age groups and without any underlying liver disease [3,44]. Furthermore, CT angiography could also help reveal the vascular architecture of FNH lesions along with the presence of a central low-density scar during the late phase, findings consistent with the centrifugal filling pattern observed in FNH [105]. 

MRI can be a useful diagnostic tool without any risk of radiation exposure, especially in women of reproductive age [3]. In MRI, pedunculated FNH may be shown as isointense to hypointense with a hypointense central scar on T1-weighted images [29]. These tumors appear isointense to hyperintense with a hyperintense central scar on T2-weighted images [29]. The central area is more commonly detected in FNH lesions with moderate to large size compared to small ones [43,47]. The presence of a pseudocapsule, hypointense and hyperintense central scars on T2- and T1-weighted images, respectively, or eventually the absence of the central scar could be atypical FNH findings that require further investigation and management [3]. Atypical features may also be observed among telangiectatic FNH cases [104]. Hepatocyte-specific contrast media, including gadoxetate disodium and gadobenate dimeglumine, could be administrated to achieve high sensitivity and specificity for diagnosing FNH [106,107]. 

Additionally, scintigraphy with technetium-99m (Tc-99m) sulfur colloid could also be used to distinguish FNH from other hepatic lesions. In nuclear imaging, these lesions are usually associated with increased focal uptake and appear as a hot spot due to their elevated concentrations of functioning Kupffer cells [90,108]. Biopsy of the lesion with histopathological analysis could be an alternative option in cases with challenges in differential diagnosis, avoiding any unnecessary surgical intervention for benign lesions. However, in practice, diagnostic inaccuracy due to biopsy sampling errors and needle tract seeding of a potential malignancy may lead to diagnostic dilemmas and controversial issues, respectively [109,110]. 

Overall, it is a matter of the utmost importance to distinguish between FNH and other focal hepatic lesions [111,112]. However, the pedunculated forms of FNH could be misdiagnosed as other exophytic tumors reported in the literature, including hepatic hemangiomas [113], hepatocellular adenomas [114], angiomyolipomas [115], lymphangiomas [116], solitary fibrous tumors [117], hepatocellular carcinomas [118], cholangiocarcinomas [119], metastases [120] and other intra-abdominal tumors, including extra-gastrointestinal stromal tumors (EGISTs) [23]. Even though pedunculated FNH lesions can appear with the imaging features described above, establishing their diagnosis can be difficult as the pedicle may not be clearly visible using the aforementioned diagnostic imaging techniques. 

## 7. Management of FNH

In most cases, FNH is asymptomatic following a silent clinical course. Exophytic lesions are commonly diagnosed as incidental findings during physical or imaging examinations. Since there is no evidence of a tendency for malignant transformation, asymptomatic FNH with typical imaging features can be safely treated conservatively without requiring any surgical intervention [3]. However, nearly 20% of all FNH patients may present with clinical manifestations [98,121]. 

Besides surgical treatment, some researchers have suggested percutaneous ablation and mostly TAE as alternative treatment options [19,30,122,123,124]. Recently, TAE was used by Nandy et al. [30] as the initial step for the management of a massive pedunculated FNH lesion causing abdominal pain. A research group presented a case of a female patient with symptomatic FNH who underwent percutaneous radiofrequency ablation of the lesion [123]. Despite the technique’s effectiveness in relieving the patient’s symptoms, a small residual nodular area was observed in the follow-up CT imaging two months later. Recently, Yao et al. [125] suggested US-guided percutaneous microwave ablation for FNH treatment due to the rapid growth of the tumor in a pediatric patient. Some authors recommended US-guided percutaneous thermal ablation as a valuable minimally invasive approach for treating FNH lesions ≤ 5 cm [126,127]. After US-guided percutaneous microwave ablation, Cheng et al. [124] reported the regrowth of a residual and partially ablated FNH lesion, which was finally treated through embolization. In fact, larger studies are required to evaluate the efficacy of ablation in FNH patients based on long-term treatment outcomes.

In addition, TAE is considered a minimally invasive alternative to surgery, particularly when surgical intervention is contraindicated. Besides avoiding the use of general anesthesia, this method also contributes to a short length of hospital stay. It can also be performed as an emergency treatment for controlling intra-abdominal bleeding caused by the potential rupture of pedunculated FNH. Meanwhile, TAE could be used as bridging therapy for FNH patients who require surgery, as it could help reduce the size of the lesions and alleviate patients’ symptoms. The main indications of TAE are FNH tumors being at anatomical locations for which access and performing surgery are difficult or technically challenging, multiple FNH lesions, and significant comorbidities. Other indications include patients who refuse surgical management or are unsuitable candidates for surgery and pediatric patients [122]. 

Common embolic agents that are used as embolization material for the treatment of FNH are polyvinyl alcohol (PVA) and gelatin sponge particles, microspheres, lipiodol emulsions, and ethanol [128]. Some researchers have suggested the combined use of bleomycin and other embolic agents, including PVA and iodized oil, for effective TAE of FNH lesions [122]. In addition to the embolic effect of iodinated oil, bleomycin plays a vital role as it affects the vascular endothelium, leading to the formation of intraluminal microthrombi and thus causing atrophy of FNH tumors via the destruction of their feeding arteries [122,129]. Complications associated with the TAE procedure include post-embolization syndrome, hepatic infarction, liver abscess formation, pleural effusion, pneumonia, and renal failure [122]. 

Surgical intervention should be considered in patients with pedunculated FNH lesions presenting with acute abdomen pain or persisting symptoms [12,29,122]. Meanwhile, several authors supported that surgical resection of FNH should also be indicated if there is any evidence is obtained of a rapid growth of the lesions using imaging techniques [12,122]. In fact, such an exophytic tumor growth could eventually lead to clinical symptoms due to the compression of the surrounding structures, including the biliary or hepatic vascular tree and the adjacent organs [3,13,30]. Interestingly, regarding rapid tumor enlargement, Perrakis et al. [12] reported that an estimated rate of tumor growth greater than 0.5 cm per year or >3 cm compared to the lesion’s initial diameter was a main indication for surgery. In patients with FNH in whom the lesion is characterized as not stable and a progressive increase in its size is detected based on medical imaging, surgical removal of the tumor is indicated. In contrast, some researchers supported the idea that surgery should not be the preferred treatment option in cases with a confident FNH diagnosis despite the increase in tumor size [130]. 

Surgical management is primarily supported in emergency settings for the complications of pedunculated FNH (as well as other FNH forms), such as intra-abdominal hemorrhage due to ruptured FNH [29,122]. Meanwhile, Li et al. [14] suggested that surgical treatment should be considered as a therapeutic option for large lesions (>5 cm in diameter) due to the potential link between the tumor size and the risk of rupture, regardless of the presence of symptoms. The authors recommended considering this possible association in treatment decision-making. Other authors supported the combination of a preoperative TAE procedure with surgery as a safe and effective treatment option for large asymptomatic lesions, given the potential risk of FNH complications [131]. 

Furthermore, other indications for surgery include the atypical localization of the tumor, challenges in differentiating exophytic FNH from other malignant liver tumors and establishing the definitive diagnosis [3,29,114,118,132]. Resection of the tumor could be performed via laparoscopy or open surgery. Surgery is considered a valuable option when treating FNH, significantly improving the clinical outcomes and quality of life, particularly for symptomatic patients [133]. Furthermore, operative treatment could aid in determining the definitive diagnosis through the pathological assessment of the resection specimen, specifically for cases with inconclusive biopsy results and diagnostic uncertainty.

To date, the benefits of laparoscopic surgery compared to open procedures are well-established. When performed by an experienced surgeon, the laparoscopic approach could offer several advantages over open tumor resection, including less perioperative blood loss, shorter length of hospital stay and rapid recovery [3,134]. During the last decade, significant technological advancements have improved the performance of such laparoscopic procedures with better outcomes, constantly moving toward an era of minimally invasive liver surgery [135].

Regarding the pediatric population, Zarfati et al. [136] suggested the surgical treatment option in cases with uncertainty of diagnosis, persistence of symptoms and significant vascular or anatomical abnormalities. In pediatric patients, establishing a definitive diagnosis, particularly in cases with atypical presentation or findings on imaging, is of utmost importance since only a limited percentage (approximately one-third) of primary hepatic tumors are benign [35,39,41]. Despite the diagnostic doubt, surgical intervention for FNH is more commonly performed among children than adults due to the more frequent presentation of clinical symptoms, even in small FNH lesions [41]. Ortega et al. [41] recommended surgery for symptomatic patients or, eventually, large asymptomatic resectable tumors. Further studies are required to evaluate the role of minimally invasive procedures compared to surgery for FNH treatment in children based on short- and long-term outcomes.

Even though the pedunculated forms of FNH are rare, they are usually associated with clinical symptoms and severe complications. Given the increased risk of the pedunculated FNH for compression of the adjacent organs and the biliary and hepatic vascular tree, hemorrhage, and infarction due to pedicle torsion, surgery is more commonly performed [24,29]. In fact, the laparoscopic approach or TAE of the feeding artery is suggested for symptomatic pedunculated FNH cases [24,29].

**Figure 3:**
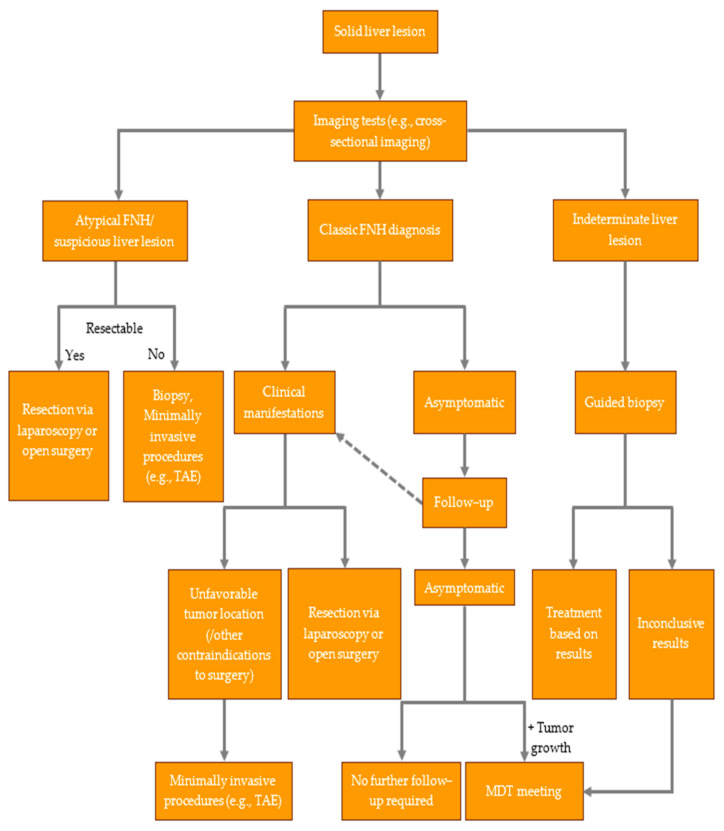
Proposed management algorithm for patients with suspected FNH. FNH, focal nodular hyperplasia; TAE, transarterial embolization; MDT, multidisciplinary team.

In guiding clinical judgement and decision-making regarding the most appropriate management for FNH patients, it is vital to better understand and weigh the risks and benefits of each treatment option. Herein, we also support the importance of multidisciplinary teams, particularly in some challenging cases, and suggest a management algorithm for patients with suspected FNH (Figure 3). According to the American College of Gastroenterology [137], pregnancy and oral contraceptive use are not contraindications among FNH patients (conditional recommendation). In fact, it is suggested that female FNH patients who continue using oral contraceptives should undergo a US examination annually for two to three years. In contrast, female patients do not require further follow-up imaging tests when they are not on oral birth control, even with a definitive FNH diagnosis (conditional recommendation). The European Association for the Study of the Liver [138] supported that follow-up might be necessary for typical FNH lesions only in patients with an underlying vascular hepatic disorder (weaker recommendation). Referring to a multidisciplinary team is strongly recommended when a patient presents with symptoms or atypical imaging features (strong recommendation).

## 8. Conclusions

FNH is a benign hepatic tumor that shows a strong female predilection. In view of all that has been mentioned so far, most cases remain asymptomatic and do not require surgical treatment. However, FNH may rarely present as an exophytic growth leading more commonly to severe complications that mandate surgical intervention. Our review demonstrates the significance of imaging examinations in establishing the definitive diagnosis of FNH. It strongly supports the idea that surgical exploration should be considered a valuable therapeutic option in selected cases, particularly when the benefits outweigh the procedure’s potential risks. Overall, all these data provide a good starting point for further research on the role of surgery, minimally invasive techniques and multidisciplinary team meetings in successfully managing FNH and other benign hepatic tumors.

## Figures and Tables

**Table 1:** Summary of studies investigating the pedunculated or exophytic FNH cases for the adult population.

Author, Year	Age	Gender	Size of Lesion	Symptoms	Procedure	Location
Bader et al., 2001 [15]	N/A	N/A	4.5 cm in diameter	+	Surgery	V
Byrnes et al., 2004 [16]	30	Female	16 × 18 cm, 3.5 × 3 cm	+	Open surgery	IVb, III
Leone et al., 2005 [17]	61	Female	6 cm in diameter	−	Follow-up	VI
Wasif et al., 2008 [18]	48	Female	3.2 × 2.4 × 2.4 cm	+	Laparoscopic Resection	IVb
Khan et al., 2011 [19]	29	Female	10 × 10 × 10 cm, Large pedunculated mass	+	Embolization & Surgery	IVb, III
Terada et al., 2012 [20]	26	Female	4 × 5 × 4 cm	−	Laparoscopic Resection	Left liver lobe
Eris et al., 2013 [13]	23	Female	7.8 × 5.5 × 5.6 cm	+	Open surgery	IVb
Dreizin et al., 2014 [21]	45	Male	Pedunculated mass	+	Laparoscopic Resection	Left liver lobe
Badea et al., 2015 [22]	29	Female	8 × 5 cm	+	Laparoscopic Resection	V
Reddy et al., 2015 [23]	35	Female	2.89 cm × 2.7 cm	+	Laparoscopic Resection	III
Zeina et al., 2016 [24]	25	Female	4.8 cm in diameter	+	N/A	II
Tsukui et al., 2017 [25]	39	Male	3.9 × 3 × 3.7 cm	−	Laparoscopic Resection	VI
Martiniuc et al., 2018 [26]	40	Female	4.2 × 5 cm	−	Open surgery (Mini- laparotomy)	III
Su et al., 2020 [27]	33	Female	8.3 × 5.9 × 5.6 cm	−	Open surgery	Left liver lobe
Navarini et al., 2020 [28]	26	Female	13 × 7 cm	+	Open surgery	Left liver lobe
Ben Ismail et al., 2021 [29]	38	Female	4.5 × 3.5 × 3 cm	−	Open surgery	III
Nandy et al., 2023 [30]	40	Female	8.2 × 14.6 × 16 cm	+	TAE & Surgery	V
Akaguma et al., 2023 [31]	35	Female	7 × 7 × 5 cm	+/−	Laparoscopic Resection	VI

FNH: focal nodular hyperplasia; N/A: not available; TAE: transarterial embolization.

**Table 2:** Summary of studies investigating the pedunculated or exophytic FNH cases for the pediatric population.

Author, Year	Age	Gender	Size of Lesion	Symptoms	Procedure	Location
Sawhney et al., 1992 [32]	12	Female	Pedunculated mass	−	Surgery	Left liver lobe
Hierro et al., 2013 [33]	13	Female	10 × 9 × 8 cm	+	Open surgery	Left liver lobe
Lee et al., 2014 [34]	7	Male	4.8 × 3 × 3.6 cm	+	Open surgery	I
	13	Female	3.1 × 4.1 × 5.5 cm	+	Open surgery	I
Koolwal et al., 2018 [35]	3	Female	5.9 cm in diameter	+	Surgery	Right liver lobe

FNH: focal nodular hyperplasia.

## Data Availability

All the data produced have been included in the present review.

## Abbreviations

FNH	Focal nodular hyperplasia
N/A	Not available
TAE	Transarterial embolization
SD	Standard deviation
IVC	Inferior vena cava
GB	Gallbladder
HSCT	Hematopoietic Stem Cell Transplantation
HUMARA	Human androgen receptor assay
mRNA	Messenger RNA
ANGPT1	Angiopoietin-1
ANGPT2	Angiopoietin-2
AFP	Alpha-fetoprotein
US	Ultrasonography
CEUS	Contrast-enhanced ultrasound
CT	Computed tomography
MRI	Magnetic resonance imaging
Tc-99m	Technetium-99m
EGIST	Extra-gastrointestinal stromal tumor
PVA	Polyvinyl alcohol
MDT	Multidisciplinary team
